# The Burden of Dengue and the Financial Cost to Colombia, 2010–2012

**DOI:** 10.4269/ajtmh.15-0280

**Published:** 2016-05-04

**Authors:** Raúl Castro Rodríguez, Gabriel Carrasquilla, Alexandra Porras, Katia Galera-Gelvez, Juan Guillermo Lopez Yescas, Jorge A. Rueda-Gallardo

**Affiliations:** Department of Economics, Universidad de los Andes, Bogotá, Colombia; Centro de Estudios e Investigación en Salud (CEIS), Fundación Santa Fe de Bogotá, Bogotá, Colombia; Fundación Santa Fe de Bogotá, Universidad El Bosque, Bogotá, Colombia; Sanofi Pasteur Latin America, Mexico City, Mexico

## Abstract

Data on the burden of dengue and its economic costs can help guide health policy decisions. However, little reliable information is available for Colombia. We therefore calculated the burden of the disease, expressed in disability-adjusted life years (DALYs), for two scenarios: endemic years (average number of cases in non-epidemic years 2011 and 2012) and an epidemic year (2010, when the highest number of dengue cases was reported in the study period). We also estimated the total economic cost of the disease (U.S. dollars at the average exchange rate for 2012), including indirect costs to households derived from expenses such as preventing entry of mosquitos into the home and costs to government arising from direct, indirect, and prevention and monitoring activities, as well as the direct medical and non-medical costs. In the epidemic year 2010, 1,198.73 DALYs were lost per million inhabitants versus 83.88 in endemic years. The total financial cost of the disease in Colombia from a societal perspective was US$167.8 million for 2010, US$129.9 million for 2011, and US$131.7 million for 2012. The cost of mosquito prevention borne by households was a major cost driver (accounting for 46% of the overall cost in 2010, 62% in 2011, and 64% in 2012).

## Introduction

The dengue virus, responsible for dengue fever (DF) and dengue hemorrhagic fever (DHF), is the most geographically widespread vector-borne infection in countries of the southern hemisphere. In recent years, a resurgence of the disease has occurred in Latin America.^1^ In 2010, Colombia had a major outbreak with more than 150,000 cases and 289 deaths, while more than a million reported cases occurred throughout Latin America in 2012, with 187,647 of these cases in the Andean subregion (Bolivia, Colombia, Ecuador, Peru, and Venezuela).^2^ In the first half of 2013, 24,116 cases of DF were reported in the Andean subregion, with 990 deaths, yielding a mortality rate of 0.5%.^3^

The high incidence of dengue disease and the associated burden^4^ represent a prominent public health problem, which is further compounded by the complexity of disease control and the factors associated with transmission (demographic, ecological, entomological, environmental, and social).^5^ Inhabitants of endemic areas may be particularly aware of the disease if they have experienced serious consequences, such as the death of a family member or neighbor.^6^,^7^

In Colombia, legislation passed in 1990 (and subsequently in 1993 and 2001) initiated a decentralization process, whereby the responsibility for top-down vector control programs devolved by the Ministry of Health and Social Protection to the states (known as departments) and municipalities. In the current setup, the ministry directly allocates funds for the programs to the departments and districts, which also use their own resources for these activities. Individuals also personally pay for certain protective measures such as repellents or mosquito nets. All these actions together incur direct and indirect costs to society and individuals. Evaluation of these costs is important to enable informed decision making and appropriate allocation of resources to the most cost-effective strategies for controlling dengue.^8^–^10^

We have previously calculated the cost per case to the health system and to the individual of DF and DHF.^11^ The cost to the health system was derived from official data sources, whereas the cost to the individual and households was based on an extensive survey of a population-based sample. However, the true cost of DF and DHF to society includes other components.^12^ In this article, we present estimates from 2010 to 2012 for the burden of the disease and the overall cost, calculated as the sum of medical costs, income lost owing to premature death, loss of productivity, and expenditure on direct, indirect, and prevention and monitoring activities for dengue infection in Colombia.

## Methods

### Epidemiological data.

Data on the incidence of DF and DHF, age of onset, and sex distribution used for calculation and modeling of the burden of disease were taken from cases notified to the National Public Health Surveillance System (Sistema Nacional de Vigilancia en Salud Pública [SIVIGILA]) for the period 2010–2012. SIVIGILA is a nationwide platform that systematically collects information of relevance to public health in Colombia at a municipal level; notification is mandatory for certain transmissible diseases, such as dengue, as well as other nontransmissible diseases, such as cancer.^13^ Deaths due to dengue infection were identified from the databases of the Colombian National Statistics Administrative Department (Departamento Administrativo Nacional de Estadística [DANE], http://www.dane.gov.co/index.php/poblacion-y-registros-vitales/nacimientos-y-defunciones/nacimientos-y-defunciones) as of 2013. Two scenarios were defined: endemic years (2011 and 2012 in the study period, where the average number of symptomatic dengue infections was calculated) and an epidemic year (referring to 2010, the year in the study period with the highest number of cases).

### Burden of disease.

We estimated the burden of disease by calculating the disability-adjusted life years (DALYs) according to the model first described by Murray.^14^ This model uses the formula shown below:

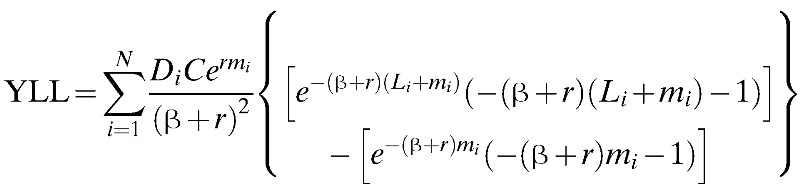


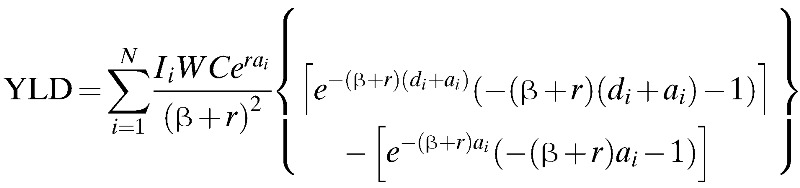


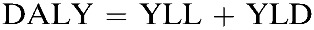
where *D* is a weighting for disability, *r* is the discount rate, *C* and β are age-weighting adjustments, *a* is the age of onset, *m* is for death, *W* is the life expectancy for each age, *I* is disease incidence, *d* is the years lost due to disability (YLD) and *L* is the years of life lost (YLL) to premature death. The subindices *i* indicate age group. The disability weighting for DF of 0.81 (0.6–0.92) was derived from a study by Meltzer and others.^15^

Using data from the 2005 DANE statistics, an exponential population growth model was derived to identify the trends in the average size and distribution of the population of Colombia in the endemic and epidemic years by age and sex and thus to estimate the YLL. The duration of disease was defined as 15 days (range = 10–21 days) based on the data from Meltzer and others.^15^

The above information was entered into the DISMOD II program, a computer application distributed free of charge by the World Health Organization (WHO).^16^ The goal of using DISMOD II is to verify that the calculated DALYs are comparable to the WHO theoretical models. DISMOD uses the downhill simplex method, which is a method of estimation of a multifactorial quantity obtained by varying factors using geometrical concepts. This allows uncertainty intervals to be calculated.^17^ We used this to obtain consistent estimators for the behavior of the disease and data on specific mortality due to dengue, the accumulated incidence and risk of dengue, together with the starting age and the average duration of the disease, separated by sex and age groups.

We obtained information on treated cases from the SIVIGILA database (individual record of health care provided). For the final calculations, a social discount rate of *r* = 0.03, an age-weighting coefficient of β = 0.04, and an age-weighting constant of *C* = 0.1648 were used.^18^

Sensitivity analyses for *K*, which is a parameter used to remove non-uniform age weights, were performed as indicated below:

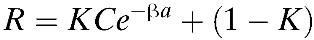
where *R* is the social value of age and *a* is age. When *K* takes the value of the weighting function of age, *K* is equal to the value used by Murray in his study^14^; when *K* is 0, all ages receive equal weight. Thus, for the analysis values, β = 0.04, *C* is assigned a value of 0.1648 and *K* a value of 0 and 1. When *K* = 0, the effect of the parameter is abolished. *K* = 1 was the value proposed by Murray and has been used in previous studies of disease burden.

A sensitivity analysis was performed using only the population at risk in an urban area living below 1,800 m above sea level (DANE data). Additional sensitivity analyses were performed with variations in age of presentation (< 1 year, 1–4 years, and then 5-year intervals up to > 80 years), duration of disease (10–21 days), and disability weighting (values between 0.60 and 0.92). In addition, a sensitivity analysis was performed using Japanese life expectancies (life expectancy of 73.81 years for men and 76.94 years for women), but otherwise DANE (http://www.dane.gov.co/index.php/estadisticas-por-tema/demografia-y-poblacion) data were used for Colombia (to investigate the impact of longer life expectancy). Finally, a sensitivity analysis was performed assuming underreporting of DF and DHF, using the underreporting factors estimated by Shepard and others^19^ (eight for DF and 1.3 for DHF).

### Costs of dengue.

The total costs related to dengue infection in Colombia were estimated as the sum of 1) treatment costs (direct medical and non-medical costs); 2) indirect costs (loss of productivity and absenteeism of both the patient and carer in the case of nonfatal episodes); 3) costs arising from lost income resulting from premature death; and 4) costs of prevention and monitoring campaigns (both by government and households, including awareness campaigns, control, and surveillance) for each period, that is, 2010, 2011, or 2012. The number of cases was taken from the SIVIGILA database for the corresponding year. All costs were calculated in pesos and converted to U.S. dollars at the average exchange rate for 2012 (1,798.23 pesos per U.S. dollars).

Total treatment costs included direct medical costs to the health system (medical appointments, treatments, and other services) and households (including co-payment of medical expenses), non-medical costs, and indirect costs to the patient and their households. For calculation of direct medical costs to the health system, information was extracted from the Individual Registries for Health Service Provision (Registro Individual de Prestación de Servicios de Salud [RIPS]). Direct and indirect medical costs to households were derived from the results of a survey of 1,483 households conducted by the Center of Economic Development Studies (Centro de Estudios sobre Desarrollo Económico) of the Universidad de los Andes, Bogotá, Colombia, in 2012 (see Castro and others^11^ for full methodological details of the survey). The estimate of the total cost under this heading corresponds to the average values per case for the period 2010–2012 multiplied by the number of cases reported by SIVIGILA in the same period (Table 1).

The estimate for the average loss of income owing to premature death had also been calculated previously.^11^ In the calculations presented here, total cost due to loss of income was calculated using the number of deaths recorded in each year of the study period (using the SIVIGILA database and the Extensive Integrated Household Survey [Gran Encuesta Integrada de Hogares conducted by the DANE] to determine the mean salary).

The costs of prevention and monitoring activities takes into account spending by national and local government bodies and households. The figure for government expenditure for these campaigns and control of the vector includes allocations made by central government to regional bodies (departments and districts) for activities concerning vector-borne diseases, and dengue in particular, according to the particular needs of each regional body. For allocations to vector-borne diseases in general, a weighting was assigned to the part corresponding to dengue based on examination of actual expenditure in each department (44.75% in 2010, 45.58% in 2011, and 44.75% in 2012). The figure also includes contracts from the Colombian Ministry of Health and Social Protection for the purchase of materials and supplies specifically for dengue and expenditure that local bodies have incurred under the terms of the Collective Action Plans (activities performed by districts to prevent, monitor, and raise awareness about dengue). The latter component was estimated by calculating an average allocation per person from a sample of municipalities and departments. The total value was then calculated by extrapolation to give a figure for the overall population at risk, which, as explained previously,^11^ was limited to approximately 24 million inhabitants of municipalities at an altitude below 1,800 m, where transmission is thought to occur.^20^

The resources allocated by the government for epidemiological surveillance, under the auspices of the Colombian National Health Institute (Instituto Nacional de Salud [INS]), comprised fees paid to staff and expenditure on technical support (transport, travel costs, and support for dengue-related events) for each of the years analyzed.

Finally, the expenditure incurred by households comprised expenditure on elements such as shutters, nets, repellents, screens, and insecticides to reduce the presence and/or restrict the intrusion of mosquitos into the household. The total cost to households for prevention was calculated by extrapolating the average costs obtained to the total population at risk, with a weighting factor assumed to be the same as used above for government spending to account for the fact that not all the above costs would apply exclusively to control the mosquito vector of the dengue virus.

To assess the uncertainty in the cost calculations, a bootstrapping analysis was performed with 10,000 repetitions, with variation in the elements recorded in the home survey and the RIPS. In addition, the impact of potential underreporting was investigated by running the calculations with underreporting factors detailed previously (eight for DF and 1.3 for DHF).^19^

## Results

In the endemic years 2011 and 2012, 3,989.7 DALYs were lost annually (83.88 per million inhabitants). In 2010 epidemic year, the total number of DALYs lost was more than 10 times greater (57,017 DALYs in total; 1,198.73 per million inhabitants) (Table 2).

In the sensitivity analysis of the population at risk in an urban area and living below 1,800 m, the burden of disease ranged from 765.4 to 1,376.5 DALYs per million inhabitants in the endemic scenario and from 9,845.9 to 13,584.2 DALYs per million inhabitants in the epidemic scenario. When the life expectancy data for Japan were used, the DALYs ranged from 201.3 to 4,120.5 per million inhabitants in the endemic scenario and from 12,347.6 to 46,781.0 per million inhabitants in the epidemic scenario. Finally, in the sensitivity analysis for underreporting, the burden of disease was 108.12 DALYs per million inhabitants in the endemic scenario and 1,222.28 DALYs per million inhabitants in the epidemic scenario.

The total treatment cost (Table 3 based on the cost per case analysis presented by Castro and others^11^) incurred by the health system for the period 2010–2012 was much higher in the epidemic year 2010 compared with the endemic years 2011 and 2012 (for 2010 versus 2011: 3.3-fold higher for outpatient DF, 1.8-fold higher for hospitalized DF, and 6-fold higher for DHF settings). Similarly, the estimated direct medical and non-medical costs incurred by households were much higher in the epidemic year compared with the endemic years. Indirect costs (arising from loss of income for both patients and caregivers) were six times greater in the epidemic year (2010) compared with the endemic years (2011 and 2012). Table 3 also shows the estimated values for loss of income through premature death. This figure, like indirect costs arising from loss of income, was more than six times greater in 2010 compared with the other two years of the study. Table 3 also shows the 95% confidence intervals calculated using the bootstrap approach described in the Methods section. To investigate the impact of underreporting, the above costs were recalculated (along with 95% confidence intervals from the bootstrap analysis) using the underreporting factors described by Shepard and others^19^ (eight for DF and 1.3 for DHF). Using these factors led to approximately a 3.5-fold increase in cost compared with the calculations with no underreporting (Table 4).

The total costs for prevention and monitoring activities did not show sharp year-on-year variations compared with other dengue-associated costs (Table 5). These costs were mainly borne by households (73% on average). Of note is that the cost of epidemiological surveillance undertaken by central government through the INS is almost insignificant (4%) compared with the total cost.

The total cost of DF (managed either in outpatient and hospital settings) and DHF, calculated by summing all the individual cost components above, is US$167.8 million for 2010, US$129.8 million for 2011, and US$131.7 million for 2012. The summary is shown in Table 6, including figures adjusted for underreported cases.

## Discussion

Information on the burden of disease and economic costs is important to allow informed policy decisions to be made and to allocate finite resources to minimize suffering due to ill health.^14^ In the case of dengue infection, the burden of disease is recognized to be high,^19^ and there are some indications that this burden may be underestimated.^17^,^21^ Moreover, the epidemiology of the disease may be changing, with factors such as globalization, climate change, and urbanization potentially contributing to a risk of more widespread epidemics.^22^ The economic burden of dengue infection is also high, although many estimates focus on the cost of medical care and neglect other cost components, such as awareness campaigns and prevention measures.^23^

In Colombia, where the disease is endemic, specific data on burden of disease on a national level are not available and general extrapolations may be subject to error in the case of dengue infection.^24^ In the case of economic costs, we recently published data on medical costs per case of DF and DHF in Colombia.^11^ Here, we extend that analysis to overall costs of the disease and, as before, our analysis included a breakdown by epidemic and endemic years.

For the burden of disease, our estimate of 83.88 DALYs per million inhabitants for endemic years (2011 and 2012) is much lower than the estimate of 1,198.73 DALYs per million inhabitants for the epidemic year (2010). Other studies have also noted large year-on-year variations generally in line with our estimates. For example, Meltzer and others^15^ found on average 658 DALYs per million inhabitants between 1984 and 1994 in Puerto Rico, with a minimum value of 145 DALYs (in 1984) and a maximum value of 1,492 DALYs (in 1994). The number of reported cases of dengue in Puerto Rico was 10-fold higher in 1994 than in 1984. In our study, more than 150,000 cases were reported in 2010 versus 33,000 cases in 2011 and 57,000 cases in 2012. In an analysis in Nicaragua, of the years 1996–2010, the annual DALYs per million inhabitants ranged from 99 in 2004, the year with lowest incidence of cases in the study period, to 805 in 2010, the year with highest incidence of cases.^25^ The figures for Latin American countries appear to be similar to those reported in other regions; for example, a recent study reported a range from 240.3 to 1,006 DALYs in Cambodia.^26^ In general, most of the burden of disease is attributed to DF rather than DHF,^25^,^26^ presumably reflecting the much higher incidence of DF. In the sensitivity analyses performed for individuals in urban areas below 1,800 m, the burden of disease was found to be much higher and in line with that reported in studies in Brazil^27^ and Thailand.^28^

One of the advantages of estimating burden of disease through DALYs is that it is possible to compare the impact of different diseases. For example, Meltzer and others^15^ concluded that the burden of dengue in Puerto Rico was similar to the burden attributed in Latin American countries to diseases such as meningitis, childhood infectious diseases (polio, mumps, pertussis, diphtheria, and tetanus), hepatitis, and malaria. Another study of neglected tropical diseases in Latin America and the Caribbean placed dengue infection fifth in terms of DALYs, behind hookworm infection, ascariasis, trichuriasis, and Chagas disease.^4^ Moreover, in the case of dengue infection, the authors recognized there are some indications that the burden may be underestimated given the potential for underreporting of the disease.^17^,^21^ Some comparisons of estimates of DALYS in Colombia are shown in Table 7.^29^

In the economic analysis shown in Tables 3 and 4, interesting findings are highlighted. Table 3 shows estimated costs of treatment and management of the disease, which are 6-fold greater for households and 3-fold greater for the health system in the epidemic year (2010) versus the non-epidemic years (2011 and 2012). A different pattern is seen in Table 5, which shows that costs relating to prevention and monitoring activities accounted for between 62% (2010) and 88% (2011) of the total cost of DF (Table 6). Our results for costs relating to prevention and monitoring activities are high compared with figures of 43% in Panama,^30^ 49% in Puerto Rico,^12^ and 39% in Thailand,^31^ and may reflect differences in the weighting given to dengue for measures aimed at mosquito control. They may also reflect the high proportion of this expense borne by households (74%). Interestingly, although overall cost increased in epidemic years as expected and in line with other studies, costs for prevention and monitoring activities show an increasing trend in the household expenses, indicating awareness in the population about dengue infection in Colombia. Moreover, government costs increased after the epidemic year but returned to the initial level the following year (2012). This may reflect the way budget allocation occurs in Colombia; when the epidemic occurred in 2010, an increased budget for prevention and control was allocated to the following fiscal year.

Finally, in the macroeconomic context of Colombia, the cost of dengue in an endemic period (2012) represented 107.61% of the budget for the Expanded Program on Immunization, 0.14% of the total national budget, and 0.036% of the gross domestic product, reflecting the considerable economic impact on a country with scant resources.

Potential limitations of the study include underestimation of both the burden of disease and costs. Our incidence data are based on entries in the SIVIGILA database where notification of cases of dengue is mandatory and so patients who seek medical care for their disease are likely to be registered in the system, assuming that DF and DHF are correctly diagnosed. This is in contrast to the study in Nicaragua reported by Wettstein and others,^25^ in which expansion factors were applied because the Ministry of Health database used in that study captured considerably fewer cases than those estimated from cohort studies.^32^ Patients who do not seek medical attention are not included in our estimate, but it can also be supposed that the burden of diseases and costs in these patients will be minimal. Although, for these reasons, the impact of underreporting on the results for costs is debatable, we nevertheless performed the cost calculations assuming the underreporting factors (Table 6) derived from a recent work that attempted to quantify underreporting.^19^ In the epidemic year, the total costs increased approximately 2-fold, whereas the increases were smaller in the endemic years, presumably mainly reflecting higher treatment costs. No sensitivity analysis was performed for possible underreporting of deaths. Although it might be expected that such events would not be underreported, recent evidence suggests that this may indeed be the case, even in places such as Puerto Rico with well-funded surveillance systems.^33^

The extent to which the household survey used for calculating costs can be considered representative was discussed in our previous analysis of medical costs per case.^11^ Overall, we considered our sample as representative of the population of patients with dengue infection in Colombia but biases could not be ruled out.

In conclusion, the high burden of disease for dengue, as measured by DALYs, is confirmed in Colombia. Similarly, the economic costs of the disease are high and are largely borne by households. This highlights the importance of considering such expenditures when studying the economic impact of the disease.

## Figures and Tables

**Table 1:** Cases of dengue in Colombia, 2010–2012

Disease classification	Setting	No. of cases (%)		
		2010	2011	2012
Dengue fever	Outpatient	107,016 (69.9)	19,366 (59.3)	33,054 (57.7)
	Hospitalized	36,404 (23.8)	11,970 (36.7)	22,719 (39.7)
Dengue hemorrhagic fever	Intensive care	9,745 (6.4)	1,303 (4.0)	1,465 (2.6)
Total		153,165	32,639	57,238

Data from Sistema Nacional de Vigilancia en Salud Pública, Bogotá, Colombia.

**Table 2:** DALYS lost per million inhabitants in the endemic and epidemic scenarios, by sex, in Colombia

Age (years)	Men			Women			Total		
	Population size	DALYs* (endemic)	DALYs* (epidemic)	Population size	DALYs* (endemic)	DALYs* (epidemic)	Population size	DALYs* (endemic)	DALYs* (epidemic)
0–4	1,815,577	340.94	2,150.57	1,707,616	363.08	1,650.82	3,523,193	351.67	1,908.35
5–14	3,604,510	102.75	2,308.24	3,396,185	153.22	1,773.47	7,000,695	127.23	2,048.81
15–29	7,222,646	81.49	1,345.59	6,960,513	55.39	1,013.06	14,183,159	68.68	1,182.39
30–44	5,703,263	71.40	1,325.24	5,946,071	25.91	1,085.65	11,649,334	48.18	1,202.95
45–59	3,319,696	18.38	768.81	3,539,977	48.50	660.88	6,859,673	33.93	726.66
60–69	1,161,649	36.33	85.15	1,279,273	13.47	14.93	2,440,922	34.35	48.35
70–79	667,696	7.74	47.07	798,468	29.02	29.44	1,466,164	19.33	37.47
≥ 80	192,682	0.09	21.28	248,923	2.14	12.56	441,605	6.88	16.36
Total	23,687,719	88.42	1,362.78	23,877,026	79.37	1,035.97	47,564,745	83.88	1,198.73

DALY = disability-adjusted life-year.

In-house calculations are based on mortality information from Departamento Administrativo Nacional de Estadística. Cases occurring are from Sistema Nacional de Vigilancia en Salud Pública, Bogotá, Colombia.

* No. of DALYs per million inhabitants.

**Table 3:** Total cost to the health system and households between 2010 and 2012 and cost of loss of income due to premature death (with no correction for underreporting)

Type of cost	Cost (US$) (95% CI*)		
	2010	2011	2012
Cost to health system†			
Outpatient DF	5,650,192 (5,586,556–5,713,360)	1,804,802 (1,753,591–1,851,990)	1,569,644 (1,438,477–1,703,801)
Hospitalized DF	8,585,571 (8,013,977–9,145,049)	4,601,103 (3,912,993–5,104,008)	5,160,365 (4,320,472–5,810,157)
DHF	14,736,394 (8,939,089–24,885,807)	2,412,136 (1,983,166–2,789,072)	2,907,075 (2,076,198–3,898,951)
Total	28,972,157 (22,539,621–39,744,215)	8,818,041 (7,649,750–9,745,069)	9,637,084 (7,835,147–11,412,910)
Cost to household			
Direct medical costs‡			
Outpatient DF	1,421,718 (1,176,112–1,692,248)	257,279 (212,833–306,235)	439,126 (363,265–522,684)
Hospitalized DF	1,268,639 (1,101,221–1,468,579)	417,141 (362,092–482,883)	791,732 (687,250–916,510)
DHF	558,397 (442,114–715,721)	74,663 (59,115–95,699)	83,946 (66,465–107,597)
Total	3,248,754 (2,719,447–3,876,548)	749,084 (634,041–884,818)	1,314,804 (1,116,980–1,546,791)
Direct non-medical costs§			
Outpatient DF	3,177,839 (2,903,072–4,466,444)	575,073 (525,350–627,300)	981,538 (896,671–1,070,679)
Hospitalized DF	1,698,782 (1,569,223–1,836,728)	558,576 (515,976–603,935)	1,060,175 (979,320–1,146,265)
DHF	610,148 (540,237–686,268)	81,583 (72,235–91,761)	91,726 (81,216–103,169)
Total	5,486,769 (5,012,532–5,989,440)	1,215,232 (1,113,561–1,322,995)	2,133,439 (1,957,207–2,320,113)
Indirect costs			
Loss of productivity (patient)	4,667,680 (3,669,908–5,821,233)	1,003,138 (778,103–1,266,680)	1,762,657 (1,364,210–2,230,539)
Loss of productivity (caregiver)	3,462,388 (2,839,031–4,192,188)	743,476 (614,549–893,959)	1,284,073 (1,063,016–1,542,116)
Total	8,130,068 (6,508,939–10,013,421)	1,746,614 (1,392,651–2,160,639)	3,046,730 (2,427,227–3,772,655)
Loss of income due to premature death	17,680,602	2,847,140	2,908,688
Total costs	63,518,350 (54,461,141–77,304,226)	15,376,111 (13,637,144–16,960,661)	19,040,745 (16,245,249–21,961,157)

CI = confidence interval; DF = dengue fever; DHF = dengue hemorrhagic fever.

Costs are derived from the survey of Centro de Estudios sobre Desarrollo Económico, prepared from Ministry of Health and Social Protection and survey of households, Registro Individual de Prestación de Servicios de Salud (RIPS; 2010–2012), RIPS sample of towns (2010–2012), and Sistema Nacional de Vigilancia en Salud Pública (2010–2012) and are based on cost per case analysis presented by Castro and others.^11^

* Calculated using a bootstrap method (see text for details).

† Includes medical appointments, treatments, and other services.

‡ Co-payment and medicines.

§ Transport, nurses' and carers' fees, lodging, food, child carers' fees, changes to living quarters, post-disease expenses, and other expenses.

**Table 4:** Total cost to the health system and households between 2010 and 2012 and cost of loss of income due to premature death (with correction for underreporting)

Type of cost	Cost (US$) (95% CI*)		
	2010	2011	2012
Cost to health system†	104,492,248 (89,271,056–129,691,203)	32,373,664 (29,343,487–34,821,992)	32,681,911 (27,658,635–37,665,162)
Cost to household			
Direct medical costs‡	16,997,645 (14,134,680–20,254,125)	3,446,663 (2,884,278–4,086,857)	5,966,190 (5,002,931–7,059,605)
Direct non-medical costs§	33,911,095 (30,979,409–37,000,886)	6,648,026 (6,081,039–7,245,800)	11,483,218 (10,509,272–12,509,813)
Indirect costs¶	52,814,507 (42,562,718–64,632,220)	10,086,761 (8,196,274–12,497,799)	17,427,649 (14,105,003–21,526,503)
Loss of income due to premature death	17,680,602	2,847,140	2,908,688
Total costs	225,896,097 (194,628,463–269,259,035)	55,402,254 (49,352,219–61,499,589)	70,467,656 (60,184,530–81,669,772)

CI = confidence interval.

Costs are derived from the survey of Centro de Estudios sobre Desarrollo Económico, prepared from Ministry of Health and Social Protection and survey of households, Registro Individual de Prestación de Servicios de Salud (RIPS; 2010–2012), RIPS sample of towns (2010–2012) and Sistema Nacional de Vigilancia en Salud Pública (2010–2012) and are based on cost per case analysis presented by Castro and others.^11^

* Calculated using a bootstrap method (see text for details).

† Includes medical appointments, treatments, and other services.

‡ Co-payment and medicines.

§ Transport, nurses' and carers' fees, lodging, food, child carers' fees, changes to living quarters, post-disease expenses, and other expenses.

¶ Loss of productivity.

**Table 5:** Total costs for prevention, awareness campaigns, control, and surveillance (prevention and monitoring activities)

Type of cost	Cost (US$) (95% CI*)		
	2010	2011	2012
Costs of prevention for households	77,303,117 (72,330,832–82,275,403)	80,941,711 (75,712,167–86,171,258)	84,921,809 (78,978,777–90,864,844)
Costs of prevention, awareness campaigns, and control for government	26,923,049	33,518,468	27,645,552
Transfers from the Ministry of Health and Social Protection	9,177,877	13,042,424	9,337,393†
Contracts	0	1,278,987	0‡
Expenditure on collective action plans	17,745,172	19,197,057	18,308,160
Cost of epidemiological surveillance	41,712	42,610	44,488
Total costs	104,267,878 (99,295,594–109,240,164)	114,502,790 (109,273,245–119,732,336)	112,611,849 (106,668,818–118,554,884)

CI = confidence interval.

In-house calculations are based on Registro Individual de Prestación de Servicios de Salud, survey of households, Ministry of Public Health and Social Protection, Sistema Nacional de Vigilancia en Salud Pública, Health Secretariats, Instituto Nacional de Salud, and Departamento Administrativo Nacional de Estadística.

* Calculated using a bootstrap method (see text for details).

† Figures calculated in pesos for December 2012 (Resolutions 404/2012 and 461; 5233/11) and converted to U.S. dollars.

‡ Information available as of September 2012.

**Table 6:** Total cost of dengue fever and dengue hemorrhagic fever

Type of cost	Cost (millions of US$)		
	2010 (million)	2011 (million)	2012 (million)
Total without correction	167.8	129.8	131.7
With correction for underreporting	330.1	169.9	183.1

In-house calculations are based on Registro Individual de Prestación de Servicios de Salud, survey of households, Ministry of Public Health and Social Protection, Sistema Nacional de Vigilancia en Salud Pública, Health Secretariats, Instituto Nacional de Salud, Departamento Administrativo Nacional de Estadística, and Shepard and others.^19^

**Table 7:** Comparison of DALYs found in the burden of disease study at the Javeriana University 2010^29^

Disease	DALYs per million inhabitants	
	Our study	Burden of disease in Colombia
Dengue		
Endemic scenario	4,000	20*
Epidemic scenario	57,017	20,000*
Cysticercosis	NA	3,000
AIDS	NA	21,000
Hypertensive heart disease	NA	131,000

AIDS = acquired immunodeficiency syndrome; DALY = disability-adjusted life-year; NA = not available.

* 2010: point estimate with 2008 data for number of deaths and number of cases from Sistema Nacional de Vigilancia en Salud Pública for 2010.
